# Myofascial and Movement Tests after Anterior Cruciate Ligament Reconstruction

**DOI:** 10.2478/hukin-2022-0052

**Published:** 2022-09-08

**Authors:** Maciej Biały, Kamil Kublin, Grażyna Brzuszkiewicz-Kuźmicka, Rafał Gnat

**Affiliations:** 1 Functional Diagnostics Laboratory, Sport-Klinika, Scanmed Sport, Żory, Poland; 2 Institute of Physiotherapy and Health Sciences, The Jerzy Kukuczka Academy of Physical Education, Katowice, Poland; 3 PhD Student at the Institute of Physiotherapy and Health Sciences, The Jerzy Kukuczka Academy of Physical Education, Katowice, Poland; 4 Faculty of Rehabilitation, Józef Piłsudski University of Physical Education, Warsaw, Poland

**Keywords:** functional performance, NEURAC® myofascial tests, Functional Movement Screen™ tests, return to sport criteria

## Abstract

Functional evaluation after anterior cruciate ligament reconstruction is one of the key points involved in decision making about the return of patients to full and unrestricted physical activity. The objective of the present study was to verify whether myofascial chain NEURAC^®^ and Functional Movement Screen (FMS™) tests can be used to detect functional differences between the operated and the non-operated extremity in patients after anterior cruciate ligament reconstruction. A total of 83 young and physically active recreational athletes (mean age: 26.9 ± 9.7 years) who underwent primary single-bundle anterior cruciate ligament reconstruction using an autogenous semitendinosus-gracilis tendon graft were evaluated between the 3^rd^ and the 4^th^ month after surgery. Subjects received a similar, standardised rehabilitation programme. Two experienced raters, blinded to the objective of this study, were involved in functional outcome data collection using myofascial NEURAC^®^ and Functional Movement Screen tests. Only two of the NEURAC^®^ tests showed significant differences in the results between the operated and the non-operated extremity: the supine bridging (mean 2.92 vs. 3.51 points, p < 0.001) and prone bridging (mean 2.76 vs. 3.67 points, p < 0.001) tests. Additionally, the summary score of all NEURAC^®^ tests significantly differed between extremities (mean 12.08 for the operated vs. 13.67 points for the non-operated extremity, p < 0.001). Myofascial tests (supine and prone bridging) in comparison with a battery of Functional Movement Screen tests seem to be more effective in detecting functional differences between the operated and the non-operated extremity at the early stage of recovery after anterior cruciate ligament reconstruction.

## Introduction

Among all types of ligament injuries, isolated tears of the anterior cruciate ligament (ACL) account for 31.5 to 38.2%, including 24% of injuries in soccer, 15% in handball players and 13% in skiers (Vauhnik et al., 2011). The incidence of a non-contact ACL tear is greater among pivoting sports athletes between 15 and 40 years of age (Barber-Westin and Noyes, 2011a; van Melick et al., 2016; Prodromos et al., 2007). In amateurs, 3% suffer from ACL injury every year, while in elite athletes this rate is close to 15% (Moses et al., 2012). Females damage their ACL 2 to 8 times more frequently than males (Myer et al., 2013; van Melick et al., 2016). In the majority of patients, an ACL tear requires surgery. In the United States alone, 125,000–200,000 ACL reconstructions (ACLR) are carried out yearly (Mayer el al., 2015). Data collected in Scandinavia show that 32–38 patients per 100,000 inhabitants undergo ACL surgery (Granan et al., 2009). Most patients qualified for surgical intervention participated in sports at a competitive level at the time of injury (Barber-Westin and Noyes, 2011; Mayer el al., 2015). Hence, criteria for returning to unrestricted physical activity or returning to sports after ACLR are constantly being discussed in the literature (Ardern et al., 2011; Boyle et al., 2016; Narducci et al., 2011; Mayer el al., 2015).

Despite this, no consensus exists as to the time interval from surgical intervention to sport readiness, the most efficient rehabilitation programme, or a standardised diagnostic protocol that will allow safe return to pre-operative training loads and reduce the risk of subsequent injuries. Many authors underline that functional evaluation after ACLR should be based on objective criteria (Barber-Westin and Noyes, 2011a, 2011b; Engelen-van Melick et al., 2013; van Melick et al., 2016). As noted by Mayer et al. (2015), present in 70% of rehabilitation protocols, the time passed after surgery is still one of the most common criteria for returning to sports or increasing training loads (Barber-Westin and Noyes, 2011a, b). Seventeen percent take advantage of additional subjective criteria; however, only 13% of protocols are based on objective evaluation surrounding the process of decision making in sports readiness (Mayer el al., 2015). Interestingly, several authors have noticed that impaired biomechanics of the lower extremity can increase the risk of ACL re-injury (Renstrom et al., 2008; van Melick et al., 2016). Moreover, Barber-Westin and Noyes (2011b) emphasise that factors such as muscle strength, joint stability, neuromuscular control and the function of the lower extremity should be considered when deciding to return to physical activity after ACLR.

Nevertheless, most of the patients show motor control deficits in the lower extremity and the core region while returning to unrestricted sports activity (Blache et al., 2017; Mayer el al., 2015; Myer et al., 2013) and only 63% reach their pre-injury sports level (Ardern et al., 2011; Blache et al., 2017; Myer et al., 2013; Paterno et al., 2014). The main feature that characterises patients after ACLR includes the side-to-side differences observed between operated and non-operated extremities during functional activities in comparison to healthy people (Blache et al., 2017; Xergia et al., 2013), for example jumping (Ernst et al., 2000; Orishimo et al., 2010), squatting (Chmielewski, 2011), lateral step-down manoeuvre (Ernst et al., 2000), stair climbing (Chmielewski, 2011) and single-leg hopping or jumping (Ernst et al., 2000; Orishimo et al., 2010; Paterno et al., 2014). While the quantity of the asymmetry may be reduced and normalised within a year after surgery in low-demand activities such as squatting (Chmielewski, 2011), it may persist for more than a year in high-demand activities such as vertical jumping (Di Stasi et al., 2013).

There are many performance-based tests used to evaluate patients’ function and side-to-side symmetry after ACLR. Many of these measures assess the combination of neuromuscular control, muscle strength and lower extremity confidence, as well as trunk control and ability to manage external loads. Most of widely performed tests are reliable and valid, for example the single-leg hop test, Y Balance test or isokinetic strength test, and they are utilised at various stages of recovery (Engelen-van Melick et al., 2013; Narducci et al., 2011). The Functional Movement Screen (FMS™) was used by Boyle et al. (2016) to evaluate adolescent patients who underwent primary ACLR 9 months postoperatively, but it is difficult to find data concerning adults in the early stages after ACLR. Recently, the NEURAC^®^ myofascial sling tests and exercises have become a popular tool for screening and treating athletes (Linek et al., 2016) or patients with musculoskeletal disorders (Park et al., 2017). However, to date, there are no data on the use of NEURAC^®^ tests in the ACLR population.

This study sought to determine whether NEURAC^®^ myofascial chain tests and FMS™ tests can be used as tools to detect motor control impairment and asymmetries between operated and non-operated extremities in adults in the early postoperative phase after ACLR. Additionally, correlations between particular NEURAC^®^ myofascial chain tests and FMS™ were evaluated.

## Methods

### Participants

A total of 83 recreational athletes (mean age, 26.9 ± 9.7 years) who underwent primary single-bundle ACLR using the autogenous semitendinosus-gracilis (STG) tendon graft performed by an experienced surgeon (over 10 years of experience in ACLR) were evaluated in this study. The ACLR was performed on the dominant (n = 40) and the non-dominant (n = 43) lower extremity. All enrolled patients complied with the inclusion criteria, which were: ACLR using the STG graft, no history of previous ACLR (both lower extremities), and no additional extra or intra-articular repairs (e.g. posterior lateral complex reconstruction or menisci repair). Subjects were recreational athletes who participated in sports on a regular basis (min. 3 times/week), the primary sport/activity at the time of the ACL injury was: soccer (37.3%), skiing (20.5%), volleyball (15.7%), martial arts (10.8%), home activities (7.2%), basketball (3.6%), ice hockey (2.4%), handball (1.2%), and tennis (1.2%). Participants did not attend any preoperative rehabilitation. After surgery, participants received a similar standardised rehabilitation program under the direction of a physiotherapist who was not associated with this study. Each patient received precise guidelines for ACL rehabilitation in the early postoperative phase, focusing on the knee range of motion, oedema management, muscle strength and proprioception drills, as well as the progression of the exercise regime. The research was approved by the local Ethics Committee and all participants gave written informed consent. Demographic characteristics of participants are presented in Table 1.

**Table 1:** Demographic characteristics of study participants.

	Study group (N = 83)
Male/female ratio	58:25
Patient age (y)^a^	26.9 ± 9.7
Height (m)^a^	175.8 ± 8.4
Weight (kg)^a^	75.7 ± 13.6
Body mass index (kg/m^2^)^a^	24.4 ± 3.3
Time interval from ACL surgery to functional assessment (months)^a^	3.4 ± 0.5
ACLR dominant/non-dominant extremity ratio	40:43

Primary sport/activity at the time of injury^b^	
Soccer	31 (37.3)
Skiing	17 (20.5)
Volleyball	13 (15.7)
Martial arts	9 (10.8)
Home activities	6 (7.2)
Basketball	3 (3.6)
Ice Hockey	2 (2.4)
Handball	1 (1.2)
Tennis	1 (1.2)

a *Values expressed as mean ± standard deviation*

b *Number of participants(% of the group)*

### Measures

**Figure 1:**
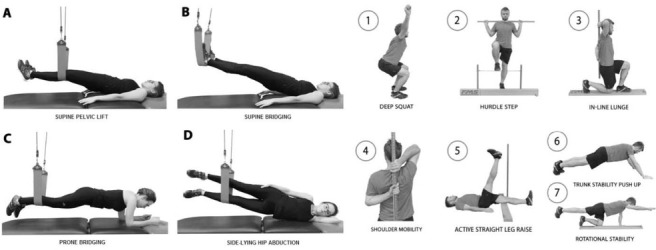
Left section: images of four NEURAC^®^ myofascial chain tests (A–D: properly performed level 3). The patient was asked to lift up to a straight body position by pressing the lower extremity into the sling. Right section: images of seven of the Functional Movement Screen™ tests (1–7).

Before the measurement all participants were asked about their well-being and health condition, especially lack of any pain symptoms within the musculoskeletal system. During the measurement, the pain was not specifically controlled using pain scales (e.g. VAS), however, participants were clearly informed to terminate the test in case of pain occurrence at any stage of NEURAC^®^ or FMS™ testing. The examination of each participants was preceded by a 10-min warm up on a bicycle ergometer, during which the rater briefly explained each step of the functional assessment procedure. First, to evaluate side-to-side differences, four basic NEURAC^®^ myofascial chain tests were implemented: supine pelvic lift (SPL), supine bridging (SB), prone bridging (PB) and side-lying hip abduction (SLHA) (Figure 1 – left section). Each of these myofascial tests has five standardised levels of difficulty. In order to pass each level, the test should be performed without provoking any pain or misalignment of the body segments. At each level, the rater evaluates the quality of movement from the starting to the end-range position, capability to maintain the end-range position for more or less than 3 s (one regular breathing cycle), and quality of movement when returning to the starting position. A level 3 score is expected for a person without any musculoskeletal problems. The right and left sides of the body are tested separately for comparison and the weight-bearing extremity is defined as the side to be tested. At each level of testing, the participant was asked to lift up the body to a straight position by pressing the lower extremity into the sling. The rater used verbal instructions during the first trial. If participants succeeded, they progressed to the next level. If they failed, the rater marked the score (the last level at which the participant succeeded). Subsequently, the rater tested the opposite side. Scoring at level 1 or 2 is described as a “weak link” (e.g. biomechanical and/or neuromuscular dysfunction). Side differences at level 3 and higher are considered to be neuromuscular imbalances (Kirkesola, 2009). In the next step of the procedure (after an approximately 5-min rest interval), the standardised FMS™ test was performed. The battery of FMS™ tests consists of seven dynamic tests to evaluate the quality of fundamental movement patterns, which are based on muscle strength, flexibility, range of motion and neuromuscular control, and to detect any significant movement dysfunction or asymmetry (Chorba et al., 2010; Minick et al., 2010). Each participant was allowed three trials for each of the seven tests [the deep squat (DS), in-line lunge (ILL), hurdle step (HS), shoulder mobility (SM), active straight leg raise (ASLR), trunk stability push-up (TS), and quadruped rotary stability (RS)] (Figure 1 – right section). Each test was scored from 0 to 3 points using specific evaluation criteria, with a total possible score from 0 to 21 (the sum of scores from seven tests) (Minick et al., 2010; Teyhen et al., 2012a). Participants scored 0 if pain occurred during the movement, 1 if they were unable to complete the movement task, 2 if they were able to complete the task but with additional compensatory movements, and 3 if they completed the task efficiently without any pain or compensatory movements. The FMS™ has been verified to be reliable (Minick et al., 2010; Teyhen et al., 2012a) and efficient (Teyhen et al., 2012b). Additionally, in this study, the number of bilateral movements in which the lower extremities were involved was recorded for detecting asymmetries between the operated and the non-operated extremity.

### Design and Procedures

This was a retrospective review of prospectively collected data from a cohort undergoing ACLR surgery. All data were collected in the early postoperative phase (3.4 ± 0.5 months) after ACLR.

Two raters blinded to the objective of this study were involved in the collection of data on the patients’ functional outcome. These raters were experienced certified physiotherapists (8 years of experience in orthopaedic rehabilitation), with 4 years of training in functional diagnosis using NEURAC^®^ and FMS™ tests. A pilot study was conducted with 12 participants in order to achieve a reliable level of agreement between raters for NEURAC^®^ myofascial chain and FMS™ tests which resulted in a weighted Kappa-Kohen coefficient of 0.75. Data processing was carried out by a third independent rater who was not informed about the objective of the study, the type of patients tested, the type of surgery, or the time interval from surgery to functional assessment.

The instruments used included the Redcord Workstation suspension and sling system device for body-weight-bearing NEURAC^®^ myofascial chain tests and a standard FMS™ Kit for functional movement evaluation.

### Statistical Analysis

Due to the ordinal character of the gathered data, non-parametric statistics were used, such as the Mann-Whitney U test and the r-Spearman correlation coefficient. The threshold of statistical significance was set at *p* = 0.05. Moreover, the numbers and percentages of participants were calculated who scored higher, equal or lower for the non-operated than for the operated lower extremity in the selected functional tests. This calculation was based on the assumption that, at the 3^rd^–4^th^ month after surgery, the operated lower extremity may show lower scores than the non-operated extremity due to its incomplete recovery.

## Results

Statistical comparison of the scores of the individual functional tests showed significant differences between the operated and the non-operated lower extremity considering NEURAC^®^ tests alone, such as the SB test (2.92 vs. 3.51 points, respectively, *p* < 0.001), PB test (2.76 vs. 3.67 points, *p* < 0.001) and the summary score of all NEURAC^®^ tests (12.08 vs. 13.67 points, *p* < 0.001). No significant differences were found for all remaining functional tests.

In the analysis of the number of participants who scored higher, equal or lower for the operated than for the non-operated lower extremity in the selected functional tests (Table 2), the tests mostly demonstrated equal scores, despite the observed lower functional capacity of the operated extremity. The only exceptions were the SB and PB tests, in which non-operated extremities were scored higher (better) in 52 (62.6%) of the cases.

**Table 2:** The number of participants who: 1) showed higher (better) scores for the non-operated (NO) than for the operated (O) lower extremity – NO > O; 2) showed equal scores for the NO and the O lower extremity – NO = O; and 3) showed lower (worse) scores for the NO than for the O lower extremity – NO < O; in the selected FMS™ and NEURAC^®^ tests.

	Test	NO > O	NO = O	NO < O
	HS	9 (10.8)	66 (79.5)	8 (9.7)
**FMS™**	ILL	5 (6.0)	75 (90.4)	3 (3.6)
	ASLR	4 (4.8)	68 (81.9)	11 (13.3)
	RS	1 (1.2)	79 (95.2)	3 (3.6)
	
	SPL	9 (10.8)	65 (78.4)	9 (10.8)
**NEURAC^®^**	SB	52 (62.6)	26 (31.3)	5 (6.1)
	PB	52 (62.6)	27 (32.5)	4 (4.7)
	SLHA	9 (10.8)	71 (85.6)	3 (3.6)

*HS – hurdle step, ILL – in-line lunge, ASLR – active straight leg raise, RS – quadruped rotary stability, SPL – supine pelvic lift, SB – supine bridging, PB – prone bridging, SLHA – side-lying hip abduction*

Several positive, weak and significant correlations between the FMS™ and NEURAC^®^ test scores were found. With regard to the operated extremity, correlations were found between the ILL and PB tests (r = 0.26), the ILL test and the summary score of the NEURAC^®^ tests (r = 0.19), the summary score of the FMS™ tests and the SB test (r = 0.25), the summary score of the FMS™ tests and the PB test (r = 0.26), and summary scores of the FMS™ and NEURAC^®^ tests (r = 0.25) (Table 3). With regard to the non-operated extremity, correlation was found only between the ILL and PB tests (r = 0.26) and the ILL test and the summary score of the NEURAC^®^ tests (r = 0.16) (Table 3).

**Table 3:** Matrix of correlations between scores of the selected NEURAC^®^ (columns) and FMS™ (rows) tests and their summary scores for the operated and the non-operated lower extremity.

Operated extremity					

Test	SPL	SB	PB	SLHA	SUMM
HS	0.14 (0.15)	0.15 (0.14)	-0.02 (0.94)	0.08 (0.20)	0.06 (0.22)
ILL	0.14 (0.17)	0.19 (0.15)	0.26 (0.01)*	0.16 (0.06)	0.19 (0.01)*
ASLR	0.06 (0.23)	0.04 (0.39)	0.15 (0.12)	0.01 (0.35)	0.08 (0.14)
RS	0.04 (0.71)	0.17 (0.15)	0.14 (0.30)	0.04 (0.90)	0.15 (0.30)
SUMM	0.16 (0.06)	0.25 (0.01)*	0.26 (0.01)*	0.15 (0.06)	0.25 (0.01)*

**Non‐operated extremity**					

HS	0.18 (0.18)	0.06 (0.77)	0.05 (0.54)	-0.06 (0.53)	0.08 (0.44)
ILL	0.11 (0.18)	-0.04 (0.84)	0.16 (0.06)	0.26 (0.02)*	0.16 (0.01)*
ASLR	0.06 (0.54)	0.13 (0.33)	-0.01 (0.94)	-0.04 (0.65)	0.07 (0.59)
RS	-0.05 (0.65)	0.10 (0.36)	0.05 (0.91)	-0.11 (0.38)	0.02 (0.96)
SUMM	0.13 (0.21)	0.15 (0.18)	0.17 (0.17)	0.00 (0.91)	0.17 (0.06)

Spearman’s r coefficients (p levels) are presented, *statistically significant, SPL – supine pelvic lift, SB – supine bridging, PB – prone bridging, SLHA – side-lying hip abduction, HS – hurdle step, ILL – in-line lunge, ASLR – active straight leg raise, RS – quadruped rotary stability.

## Discussion

The aim of the present study was to determine whether the chosen NEURAC^®^ and FMS™ tests are appropriate tools to detect motor control impairment and functional asymmetries between operated and non-operated extremities in the early postoperative phase after ACLR. To the best of our knowledge, this is the first comparison of NEURAC^®^ tests with FMS™ functional movement tests in the ACLR population. The most important finding was that NEURAC^®^ tests, such as SB (operated extremity 2.92 points vs. non-operated 3.51 points) and PB (2.76 vs. 3.67 points) tests, as well as the NEURAC^®^ summary score (12.08 vs. 13.67 points), were able to detect side-to-side differences between the operated and the non-operated extremity. None of the FMS™ tests, which were originally developed to evaluate side-to-side motor control imbalances (HS, ILL, ASLR, RS) between lower extremities, showed significant differences.

Moreover, we assumed that, at the selected post-ACLR treatment stage, the operated extremity would show worse functional outcomes due to its incomplete recovery. In accordance with this, we found that SB and PB tests showed higher results for the non-operated extremity in 62.6% of the patients. In contrast, all of the aforementioned FMS™ tests demonstrated equal scores between extremities in the majority of the cases, for example, in 90.4% of cases for the ILL test and in 81.9% for the ASLR test.

All of the NEURAC^®^ tests are intentionally designed to evaluate side-to-side differences and use progressive body-weight loads to detect asymmetries. It is well known that use of the STG type of graft promotes significant asymmetries in quadriceps and hamstring muscle strength of the operated extremity as compared to the non-operated extremity, both in the early and late post-ACLR recovery stage (Ithurburn et al., 2018; Rogowski et al., 2019). Therefore, it seems crucial to employ a diagnostic tool that is safe at each stage of recovery after ACLR and also allows detection of side-to-side asymmetries. The nature of both SB and PB NEURAC^®^ tests reflects these specific requirements. The SB and PB tests place body weight on the addressed muscle groups (SB – hamstring muscles; PB – quadriceps femoris) with constant underlying core control. This may explain the significant results between the operated and the non-operated extremity and the higher number of worse scores for the operated extremity observed in these particular tests (62.6%).

Attempts to screen ACLR functional outcomes using the FMS™ tests provide contradictory data. Based on the results of this study, there is no significant difference between the operated and the non-operated extremity for the chosen FMS™ tests. The percentage of lower scores for the operated extremity is low, and ranges from 1.2 to 10.8%. Similar results were reported by Mayer et al. (2015) who evaluated ACLR patents 6 months after surgery and found no significant difference in the FMS™ test results between the patients who were clinically cleared for return to sporting activities without restriction (total FMS™ score: 12.7 ± 2.9) and those who were not (total FMS™ score: 12.8 ± 2.7). Originally, the FMS™ was developed to assess functional movement on the basis of sufficient muscular strength and proper motor control. However, based on our results, we can conclude that FMS™ tests for the trunk and lower region of the body (HS, ILL, ASLR, RS) are not specific enough to detect functional side-to-side asymmetries in ACLR patients 3 months after surgery. The higher scores of these tests may lead to overestimation of the total FMS™ score. In our research, the mean total score of FMS™ was 14.7 ± 2.4, showing better functional outcomes in our group compared to previous data in ACLR patients (Mayer et al., 2015). The use of FMS™ in postoperative testing after ACLR should be followed by the use of additional tools to provide more functional details concerning patients after ACLR.

To date, there is little data describing correlations between FMS™ and NEURAC^®^ tests. Linek et al. (2016) found that an 8-week taining program based on NEURAC^®^ exercises (employing motor tasks similar to NEURAC^®^ tests) in young male volleyball players positively influenced FMS™ results (more than 90% of the subjects scored ≥ 17 points in FMS™ after the training period), which may be important from the perspective of injury prevention in this group of athletes. In our study, NEURAC^®^ tests and FMS™ tests showed almost no significant correlations, with some exceptions. In the operated extremity, the outcomes of the ILL FMS™ test were significantly related to the lower values of the PB test (both tests are asymmetrical and engage the gluteal muscles) and the total score of NEURAC^®^ (weak positive correlations of 0.26 and 0.19, respectively). Additionally, we found a significant, weak, positive relationship between the total FMS™ score and two NEURAC tests, SB (*r* = 0.25) and PB (*r* = 0.26). In the authors’ opinion, these correlations can be considered as a random outcome.

In the non-operated extremity, we noticed two weak, positive and significant correlations: the ILL test was positively correlated with the SLHA (*r* = 0.26) and the NEURAC^®^ total score (*r* = 16). The correlation between the ILL and SLHA might be explained by the nature of these movement tasks since both tests evaluate lower extremity motor control based on gluteus medius strength as well as hip, pelvis and trunk control/stability.

There are several limitations of this study. First, although the two utilized screening methods evaluate similar functional features such as motor performance based on trunk control, they are performed in two different environments: FMS™ – on a stable surface, and NEURAC^®^– in unstable suspension. Second, due to the time of enrolment we were unable to collect preoperative FMS™ and NEURAC^®^ outcomes, which would have provided a convenient baseline measurement to compare to changes in functional outcomes over time. In addition, due to the small number of female participants we did not analyze intergender differences and our study was conducted only on generally young, physically active recreational athletes, thus the results cannot be generalized (e.g. to a high-level athletes population).

Clinical application of the present results could be of importance for the optimal loading progression in physiotherapeutic protocols after ACLR, which is novel information in the professional field. In the outlined group of patients, the exclusive utilization of the FMS™ tests in the early phase of the recovery can potentially lead to overestimation of the subjects’ functional performance and expose them to excessive loads during treatment. This, in turn, decides on the safe application of the therapy. Additionally, advantages of the NEURAC^®^ SB and

PB tests are: high simplicity, low financial costs and time consumption. Moreover, these tests detect lower extremity asymmetry, which constitutes the critical factor leading to re-injuries after ACLR.
